# A Case of Waldenstrom’s Macroglobulinemia Presenting as Worsening Neuropathy

**DOI:** 10.7759/cureus.91425

**Published:** 2025-09-01

**Authors:** Meher Binte Ali, Zunera Huda, Marcus Edward Hendricks, Kathryn Kline, Danish Jilani

**Affiliations:** 1 Internal Medicine, University of Maryland Medical Center, Baltimore, USA; 2 Internal Medicine, Dow University of Health Sciences, Karachi, PAK; 3 Internal Medicine, American University of Antigua, Osbourn, ATG; 4 Oncology, University of Maryland Medical Center, Baltimore, USA

**Keywords:** deymyelinating neuropathy, monoclonal gammopathy, paraproteinemia, peripheral neuropathy, waldenstrom’s macroglobulinemia

## Abstract

Waldenstrom’s macroglobulinemia (WM) is a rare lymphoplasmacytic lymphoma characterized by proliferation of monoclonal IgM-secreting B-cells, typically presenting with anemia, hepatosplenomegaly, and lymphadenopathy. We describe a case of WM manifesting as progressive neuropathy, initially attributed to degenerative spinal disease, for which the patient underwent treatment without clinical improvement. Further evaluation revealed a monoclonal M-spike on serum protein electrophoresis (SPEP) and endoneural IgM deposition on sural nerve biopsy. Bone marrow biopsy demonstrated lymphoplasmacytic lymphoma with MYD88 L265P mutation. The patient underwent six cycles of bendamustine-rituximab with partial neurological stabilization and a significant decline in IgM levels. However, neuropathy subsequently recurred, necessitating the initiation of zanubrutinib. This case highlights the diagnostic complexity of paraproteinemic neuropathy and emphasizes the importance of considering WM in the differential diagnosis of atypical neuropathy to ensure timely intervention and mitigate irreversible nerve damage.

## Introduction

Waldenstrom’s macroglobulinemia (WM) is a variant of lymphoplasmacytic lymphoma (LPL), characterized by infiltration of lymphoplasmacytic cells in bone marrow and overproduction of IgM in the serum. It is a rare B-cell malignancy with an incidence of 4.2 per million persons per year, occurring more commonly in older, White male individuals [1]. The disease typically follows an indolent course and presents with symptoms of anemia, thrombocytopenia, lymphadenopathy, hepatosplenomegaly, and hyperviscosity [1]. The pathogenesis involves clonal B-cell proliferation driven primarily by MYD88 L265P-mediated activation of the nuclear factor kappa B (NF-KB) signaling pathway, resulting in prolonged survival of malignant cells [2]. Diagnosis is established by the detection of monoclonal IgM-kappa protein alongside > 10% clonal LPL cells in the bone marrow [1]. These lymphoplasmacytic cells commonly express the B-cell markers CD19 and CD20. Molecular testing, particularly for *MYD88* and *CXCR4* mutations, may further support the diagnosis and guide therapeutic decisions. The MYD88 L265P mutation is present in approximately 90% of patients, while *CXCR4* mutations occur in about 30% [1].

Up to 25% of patients with WM may present with peripheral neuropathy as the initial or even sole manifestation of the disease [3]. While the underlying mechanisms remain incompletely understood, several hypotheses have been proposed, including the deposition of monoclonal IgM proteins, nerve ischemia due to hyperviscosity, direct nerve infiltration by malignant cells, and IgM autoantibodies against myelin-associated glycoprotein (MAG) and gangliosides [3]. To date, only a limited number of cases of WM presenting with neuropathy have been reported in the literature, particularly when neuropathy is the sole or misleading initial presentation [4-6].

In this report, we discuss the case of a patient who presented with neuropathy, initially attributed to his neurodegenerative spinal disease, who was then eventually diagnosed with WM. This report highlights the diagnostic challenges and underscores the importance of considering WM in the differential diagnosis of atypical or refractory neuropathy.

## Case presentation

A 68-year-old male patient presented with a three-year history of progressive numbness and weakness. His symptoms began with numbness, tingling, and weakness in the right foot, which gradually involved the entire right leg. Soon after, he developed similar deficits in the right arm with biceps atrophy. Cervical spine magnetic resonance imaging (MRI) was performed, which revealed severe foraminal stenosis at the right C6-C7 level. He underwent anterior cervical discectomy and fusion (ACDF) at C5-C6 and C6-C7. However, despite surgical intervention, his symptoms continued to deteriorate.

After a few months, the patient developed numbness and weakness of the left foot and leg with associated atrophy of the left thigh muscles. The patient also reported occasional shooting pain radiating down his right arm and left leg, and slight back pain. A neurological examination performed at the time demonstrated decreased sensation and hyporeflexia in the affected regions. Repeat spinal MRI scans were performed, which demonstrated unchanged cervical findings with additional foraminal stenosis at L4-L5 levels due to pseudo-disc protrusion. Electromyography (EMG) and nerve conduction studies (NCV) were performed in June 2022, which revealed sensorimotor neuropathy. At this point, the patient’s symptoms were suspected to be due to a combination of cervical and lumbar spine disease. The patient was managed symptomatically, and a course of physical therapy was initiated; however, despite the interventions, his symptoms continued to worsen

About six months later, in January 2023, the patient presented with further aggravated symptoms, thus prompting a follow-up MRI of the spine. Imaging showed an enhanced left L3 nerve root with a bulbous configuration at the foraminal level. EMG was done, which demonstrated multifocal, mixed demyelinating and axonal neurogenic process affecting the right biceps and left vastus lateralis, and mild focal median neuropathy at the right wrist with sensory involvement and demyelinating features. The EMG and NCV findings are shown in Tables 1, 2. Due to the stepwise nature of symptoms and MRI and EMG findings, an autoimmune multifocal neuropathy was suspected, and intravenous immunoglobulin (IVIG) therapy was initiated. The induction phase consisted of IVIG at 35 g daily for four consecutive days, followed by initiation of maintenance therapy three weeks later at 35 g daily for two days. The maintenance dose was repeated every three weeks, continuing for a total duration of three months over six cycles. Despite the therapy, the patient did not demonstrate significant clinical improvement in symptoms.

**Table 1:** Needle electromyography studies (January 2023) Abnormal values are marked with an asterisk (*) * Right biceps with moderate presence of positive waves and fibrillation potentials with associated small motor units with moderately reduced recruitment pattern ** Right vastus lateralis with reduced recruitment pattern and mildly large amplitude motor units. Spontaneous activity is reported as presence/absence of fibrillation potentials (Fib) and positive sharp waves (P wave). MUAP: motor unit action potential; ↓: reduced; ↑: increased

Muscle tested	Spontaneous activity (Fib / P wave)	Recruitment	MUAP morphology (amplitude/duration/poly)
Right deltoid	Normal	Normal	Normal
Right biceps	Fib 2+, P wave sustained*	↓↓ (−2)*	↓ amp (−1), normal duration, normal poly*
Right triceps	Normal	Normal	Normal
Right extensor digitorum communis	Normal	Normal	Normal
Right flexor carpi radialis	Normal	Normal	Normal
Right first dorsal interosseous	Normal	Normal	Normal
Left vastus lateralis	Normal	↓↓ (−2)**	↑ amplitude (1+), normal duration, normal poly**
Left tibialis anterior	Normal	Normal	Normal
Left peroneus longus	Normal	Normal	Normal
Left gastrocnemius (medial head)	Normal	Normal	Normal
Left flexor digitorum longus	Normal	Normal	Normal

**Table 2:** Nerve conduction study findings (January 2023) Abnormal values are marked with an asterisk (*) * Mild focal median neuropathy at wrist with sensory demyelination ** Left peroneal motor conduction with absent response NR: no response. APB: abductor pollicis brevis; ADM: abductor digiti minimi; EDB: extensor digitorum brevis; Tib Ant: tibialis anterior; AH: abductor hallucis; R: right; L: left

Nerve	Motor Responses			Sensory Responses		
	Amplitude (mV)	Velocity (m/s)	Site of recording	Amplitude (µV)	Velocity (m/s)	Site of stimulation
Median (R)	Wrist: 10.3 Elbow: 9.9 Axilla: 8.9 Site 4: 6.1	Elbow: 54.7 Axilla: 68.6 Site 4: 63.2	ABP	3.2*	45.8*	Wrist- Digit II
Ulnar (R)	Wrist:10.6 Elbow: 10.4 (below) 9.1 (above) Axilla: 8.7 Erb’s point: 6.2	Elbow: 63.2 (below) 37.6 (above) Axilla: 64.0 Erb’s point: 56.3	ADM	11.1	53.8	Wrist- Digit V
Radial (R)	-	-		10.9	54.9	Forearm- Thumb
Peroneal (L)	NR**	-	EDB	5.8	40.3	Lateral leg – Ankle
	Fib Head: 1.8 Knee: 2.1	Knee: 36.0	Tib Ant	-	-	-
Tibial (L)	Ankle: 4.7 Knee: 5.0	Knee: 42.8	AH	-	-	-
Sural (L)	-		-	4.4	42.0	Lateral leg - Ankle

Given the continued clinical deterioration, detailed laboratory investigations were performed (Table 3). Serum protein electrophoresis (SPEP) revealed a 0.9 g/dL M spike with immunoelectrophoresis showing IgM kappa and lambda elevation. Quantitative immunoglobulin levels were also performed and were notable for IgG 379 mg/dL, IgM 1500 mg/dL, and IgA 51 mg/dL. Despite such elevated IgM levels, symptoms of hyperviscosity, such as visual changes, headache, or bleeding, were absent. Blood urea nitrogen (BUN) and estimated glomerular filtration rate (eGFR) were only mildly altered, which made overt kidney failure unlikely. Due to the elevated IgM with worsening neurological symptoms, there was an increasing concern for a lymphomatous process, prompting the decision to perform a left sural nerve biopsy.

**Table 3:** : Laboratory investigations. WBC: white blood cells; CO2: carbon dioxide; EGFR: estimated glomerular filtration rate; SPEP: serum protein electrophoresis; FTA-ABS: fluorescent treponemal antibody-absorption; ELISA: enzyme-linked immunoassay

Test	Result	Reference range
Leukocyte count (WBC) (x10^9^/L)	6.2	4.5-11.0
Hemoglobin (g/dL)	15.0	Male: 14-17; Female: 12-16
Hematocrit (%)	44.7	Male: 41-51; Female: 36-47
Platelets (x10^9^/L)	429	150-350
Sodium (mmol/L)	138	136-145
Potassium (mmol/L)	4.2	3.5-5.0
Chloride (mmol/L)	99	98-106
Calcium (mg/dL)	9.5	9-10.5
Total CO₂ (mmol/L)	29	21-30
Blood Urea Nitrogen (mg/dL)	22	8-20
Creatinine (mg/dL)	0.93	0.7-1.3
EGFR (mL/min/1.73 m²)	88	>90
Total Protein (g/dL)	7.6	6.0-7.8
Albumin (g/dL)	4.3	3.5-5.5
Aspartate aminotransferase (AST) (U/L)	39	17-59
Alanine aminotransferase (ALT) (U/L)	30	0-49
Bilirubin Total (mg/dL)	1.1	0.3-1.2
Alkaline phosphatase (U/L)	86	36-92
Lactate dehydrogenase (U/L)	172	120-246
C-reactive Protein (mg/dL)	<0.5	0.0-0.8
Alpha 1 (%)	4.3	2.9–5.2
Alpha 1 Globulin Fraction (g/dL)	0.3	0.1-0.3
Alpha 2 Globulin Fraction (g/dL)	0.6	0.6-1.0
Beta Globulin (g/dL)	0.8	0.7-1.2
Gamma Globulin Fraction (g/dL)	1.6	0.7-1.6
Vascular endothelial growth factor (pg/mL)	<31	31-86
Immunoglobulin G (IgG) (mg/dL)	379	700-1600
Immunoglobulin M (IgM) (mg/dL)	1500	40-230
Immunoglobulin A (IgA) (mg/dL)	51	70-400
SPEP M-spike (g/dL)	0.9	Not detected
Blood Vitamin B1 (Thiamine) (nmol/L)	104	70-200
Blood Vitamin B12 (pg/mL)	312	239-931
FTA-ABS	Nonreactive	Nonreactive
Hepatitis C Antibody	Negative	Negative
LYME Antibody ELISA	<0.90	<0.90: Negative; 0.91–1.09: Equivocal; ≥1.10: Positive

The biopsy revealed endoneural IgM deposition, consistent with paraproteinemic neuropathy, thereby raising clinical suspicion for WM and warranting further evaluation with a bone marrow biopsy, which revealed 30% involvement of the bone marrow by a low-grade B cell lymphoma with plasmacytic differentiation and a MYD88 L265P mutation. CXCR4 mutations and increased plasma cells were not detected. Peripheral blood flow cytometry was also performed and was negative for a monotypic B-cell population, increased blasts, or aberrant NK/T cells. A skeletal survey was conducted with no evidence of lytic lesions. The symptomatic peripheral neuropathy alongside the significant infiltration of B-cells in bone marrow strongly favored the diagnosis of WM.

Combination chemotherapy with rituximab (375 mg/m^2^) and bendamustine (90 mg/m^2^) was initiated to prevent further neurological deterioration. Following six cycles of drug therapy over a six-month period, the patient presented for a follow-up evaluation. Repeat SIEP and immunoglobulin levels were performed, revealing a significant decline in IgM levels (600mg/dL). Clinical improvement was noted during and shortly after chemotherapy, with stabilization of muscle weakness; however, in the ensuing months, his symptoms progressively worsened. He exhibited significant weakness and numbness in the left lower extremity with persistent motor deficits in the right upper limb necessitating further intervention with zanubrutinib (320 mg daily), on which he remains currently.

The entire clinical timeline is summarized in Table 4 for clarity.

**Table 4:** Chronological summary of clinical course, key investigations, and interventions ACDF: anterior cervical discectomy and fusion; EMG: electromyography; NCS: nerve conduction studies; MRI: magnetic resonance imaging; IVIG: intravenous immunoglobulin; SPEP: serum protein electrophoresis; IgM: immunoglobulin M; WM: Waldenström macroglobulinemia

Timeline	Clinical Finding	Key Investigation	Intervention/Outcome
2019 - 2022	Gradually progressive bilateral distal weakness and numbness (sensorimotor neuropathy)	MRI cervical spine: severe C6-C7 and L4-L5 foraminal stenosis	ACDF at C5-6 and C6-C7 → No symptomatic improvement
June 2022	Ongoing symptoms	EMG/NCS: sensorimotor polyneuropathy of bilateral lower extremities	Symptomatic management and initiation of physical therapy
January 2023	Worsening distal weakness and numbness with sensory deficits	MRI: L3 nerve root enhancement; EMG: multifocal demyelinating features → Autoimmune neuropathy suspected	IVIG therapy → No symptomatic improvement
February 2023 - October 2023	Ongoing symptoms	Elevated serum IgM levels; SPEP: M-spike	-
November 2023	Ongoing symptoms	Sural nerve biopsy: endoneural IgM immunopositivity; Bone marrow biopsy: 30% B-cell lymphoma with MYD88 L265P mutation → WM diagnosed	Combination chemotherapy with Rituximab and Bendamustine initiated
January 2025	Initial symptomatic improvement and fall in IgM levels, followed by gradual deterioration	-	Zanubrutinib initiated

## Discussion

WM is a rare lymphoproliferative disorder characterized by the proliferation of monoclonal IgM-producing B cells in the bone marrow. It typically follows an indolent course with clinical features of cytopenia, lymphadenopathy, hepatosplenomegaly, and hyperviscosity symptoms [1]. Neurological involvement, although less common, may be the initial or even sole disease manifestation and includes peripheral neuropathy, hyperviscosity syndrome, and Bing-Neel syndrome [3,7]. These symptoms are hypothesized to occur due to IgM deposition in nerves, direct tumor infiltration, and the presence of anti-MAG and anti-GM antibodies [3]. Up to 25% of patients with WM may present with peripheral neuropathy at the time of diagnosis, presenting as chronic symmetric sensory polyneuropathy, diminished vibration and pinprick sensations, and gait disturbances [3,8]. These symptoms often overlap with more prevalent conditions such as chronic demyelinating polyneuropathy (CIDP) and neurodegenerative diseases, leading to incorrect or delayed diagnosis.

**Figure 1:**
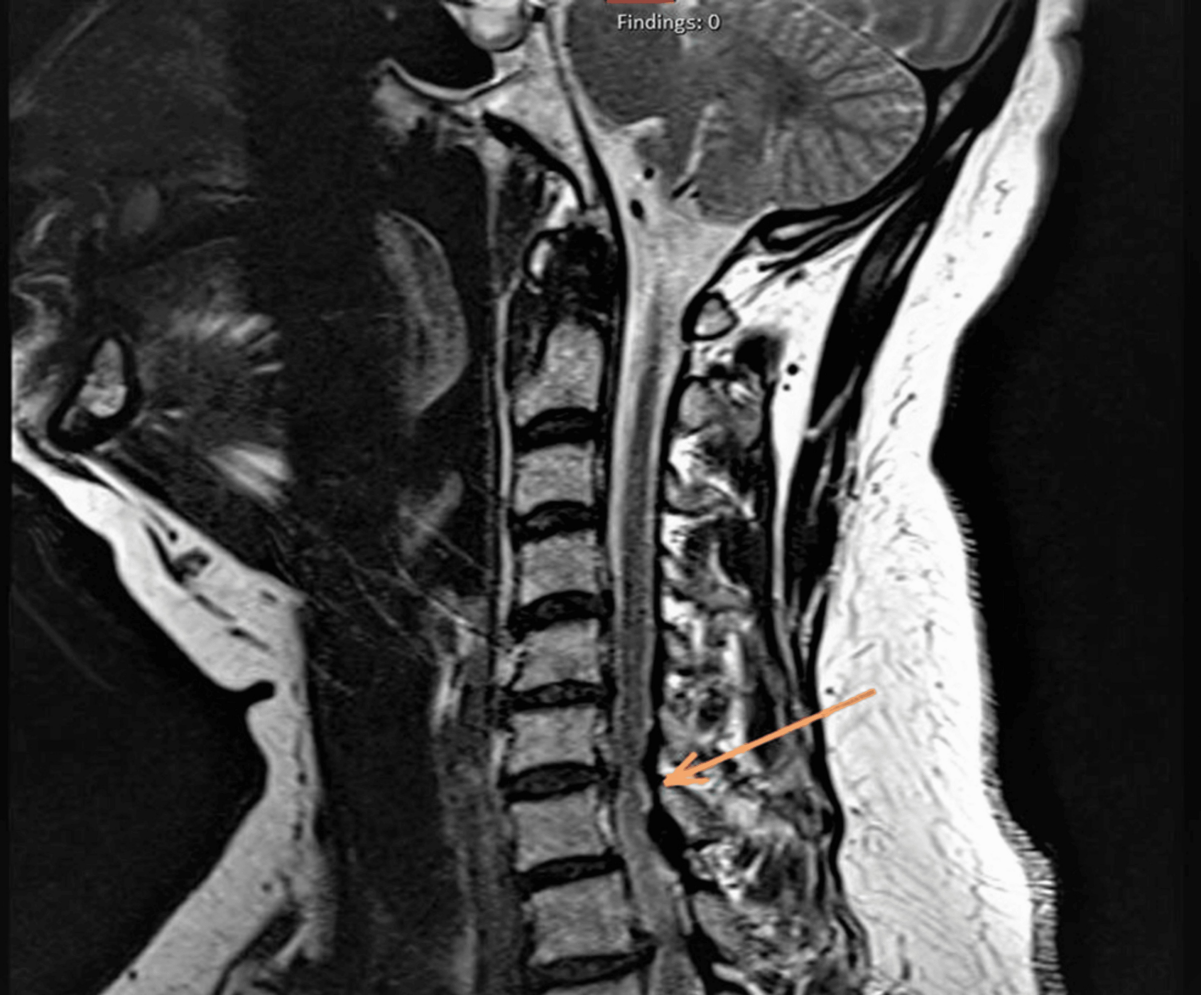
Representative image of MRI cervical spine showing severe foraminal stenosis at C6–C7 Image Source: Wasey et al., 2022 [11]; under the Creative Commons Attribution 3.0 Unported licence (https://creativecommons.org/licenses/by/3.0/)

**Figure 2:**
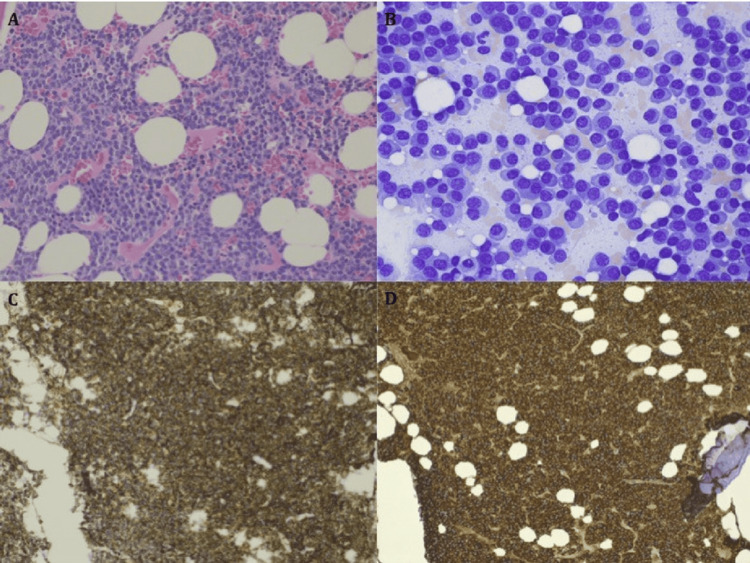
Representative images of bone marrow biopsy. (A) Hypercellular marrow with extensive sheet-forming plasma cell infiltration; (B) Higher magnification demonstrating extensive plasma cell infiltration with few lymphocytes; (C) Immunohistochemistry demonstrating marked CD138-positive population; (D) Immunohistochemistry demonstrating lambda-restricted population. Image Source: Uminski et al. [12]; under the terms of the Creative Commons Attribution Non-Commercial 4.0 International License (http://creativecommons.org/licenses/by-nc/4.0/)

Our patient presented with chronic, asymmetric polyneuropathy involving the upper and lower extremities. Initial imaging showed cervical foraminal stenosis, which led to a diagnosis of neurodegenerative disease; however, the lack of clinical improvement following ACDF and the subsequent progression of symptoms to bilateral, symmetric limb involvement raised the possibility of an alternate pathology. Further workup with EMG and NCS demonstrated demyelinating and mixed axonal features, prompting the consideration of an autoimmune pathology such as CIDP, for which IVIG therapy was initiated. CIDP is an acquired, immune-mediated, chronic demyelinating neuropathy characterized by symmetrical motor and sensory deficits and requires classic demyelinating findings in at least two motor nerves for diagnosis [9,10]. In our patient, the asymmetric distribution of symptoms, limited and multifocal demyelination, and the lack of symptomatic improvement after multiple cycles of IVIG argued against CIDP. Representative images of MRI cervical spine showing severe foraminal stenosis at C6-C7 and bone marrow biopsy are shown in Figures 1, 2, respectively.

Further evaluation showed elevated serum IgM (1500 mg/dL) and an IgM-Kappa M-spike on SPEP with IFE. These findings raised suspicion for an underlying plasma cell dyscrasia with WM, IgM-monoclonal gammopathy of undetermined significance (MGUS), and IgM-related disorders (amyloid light-chain (AL) amyloidosis, cryoglobulinemia, POEMS (Polyneuropathy, Organomegaly, Endocrinopathy, M-protein, and Skin changes) syndrome, and CANOMAD (Chronic Ataxic Neuropathy, Ophthalmology, IgM paraprotein, Cold Agglutinins, and Disialosyl antibodies) syndrome) being the top differentials. Bone marrow biopsy revealed lymphoid infiltration (30%) with plasmacytic differentiation and a positive MYD88 L265P mutation; thus, a diagnosis of WM was made. This is consistent with the criteria proposed by the International Workshop on WM (IWWM) requiring the presence of monoclonal lymphoplasmacytic cells on bone marrow biopsy and monoclonal IgM in serum for the diagnosis of WM [13]. The IWWM also recommends the assessment of the MYD88 L265P mutation as a critical diagnostic adjunct in confirming diagnosis [13].

When diagnosing WM-associated neuropathy, it is essential to consider and systematically rule out other entities with overlapping features. IgM-MGUS was a key differential and is defined as asymptomatic serum IgM paraproteinemia (< 3000 mg/dL) and < 10% bone marrow infiltration [14]. Although our patient’s IgM level fell within the MGUS range, the presence of unequivocal lymphoid infiltration and symptomatic presentation strongly favored the diagnosis of WM. Another important consideration was the spectrum of IgM-related disorders, which are characterized by clinical manifestations attributable to the IgM paraproteinemia in the absence of overt lymphoma. These include amyloid light-chain (AL) amyloidosis (typically presenting with renal or cardiac involvement), cryoglobulinemia (often associated with vasculitic features and cold-induced symptoms), POEMS (Polyneuropathy, Organomegaly, Endocrinopathy, Monoclonal protein, and Skin changes) syndrome, increased vascular endothelial growth factor (VEGF) levels, or CANOMAD (Chronic Ataxic Neuropathy, Ophthalmoplegia, IgM M paraprotein, Cold Agglutinins, and Disialosyl antibodies) [15,16]. In our case, the absence of these hallmark features on clinical evaluation and diagnostic testing made these entities unlikely.

Current guidelines by IWWM identify peripheral neuropathy attributable to WM as an indication for initiating therapy [17]. Recommended first-line options include rituximab-based regimens, including rituximab monotherapy, rituximab-bendamustine (BR), and dexamethasone-rituximab-cyclophosphamide (DRC) [17]. Our patient elected to proceed with the BR regimen due to its time-limited treatment course. However, the progressive decline after the initial clinical improvement prompted the initiation of zanubrutinib. Zanubrutinib is a BTK inhibitor; BTK plays a key role in signal transduction through the MYD88-driven NF-KB pathway, promoting the survival of neoplastic cells. Emerging studies have shown the promising efficacy of BTK inhibitors, including zanubrutinib and Ibrutinib, in patients with refractory/ relapsed WM, such as in our case [17-20].

Early recognition and treatment of WM-related neuropathy are crucial to prevent irreversible nerve injury. A multidisciplinary approach further facilitates better outcomes. In our case, neurology was involved early to evaluate the cause of neuropathy and to facilitate the initiation of the IVIG trial. Following the diagnosis of WM, oncology was consulted to manage the underlying disease. The physical therapy team was also engaged throughout the patient’s course to support recovery and rebuild muscle. Although full recovery of neurological function is often limited, treatment can stabilize the disease and prevent further progression.

There were a few limitations to our study. No standard neurological score was used to assess response to therapy, which can be important in providing objective data. Due to institutional limitations, images could not be provided, which may limit individual interpretation of data by the readers.

## Conclusions

This case report highlights WM as a rare but important cause of chronic progressive neuropathy. Linking polyneuropathy to underlying hematological malignancies remains a diagnostic challenge, as illustrated by our patient in whom early symptoms were misattributed to spinal pathology and empiric IVIG therapy provided no benefit. Our case underscores the need for vigilance and the importance of a multidisciplinary approach in patients with unexplained polyneuropathy, particularly when structural and autoimmune causes are excluded. Despite their clinical significance, paraproteinemic neuropathies are often misdiagnosed. Given the variable initial presentation of WM, measurement of IgM levels and a bone marrow biopsy are key to diagnosis and should be incorporated early in diagnostic workup. Prompt recognition and tailored therapies are crucial to prevent irreversible complications and improve patient outcomes. Rituximab-based regimens remain the first-line treatment of choice for WM; however, BTK inhibitors should be considered in cases of relapse.
